# Involvement of Cox-2 in the metastatic potential of chemotherapy-resistant breast cancer cells

**DOI:** 10.1186/1471-2407-11-334

**Published:** 2011-08-04

**Authors:** Ju-Hee Kang, Ki-Hoon Song, Kyung-Chae Jeong, Sunshin Kim, Changsun Choi, Chang Hoon Lee, Seung Hyun Oh

**Affiliations:** 1 Division of Cancer Biology, National Cancer Center, Ilsan-ro 323, Ilsandong-gu, Goyang-si, Gyeonggi-do 410-769, Republic of Korea; 2 Department of Food and Nutrition, College of Human Ecology, Chung-Ang University, 72-1, Nae-ri, Daeduck-myun, Ansung, Gyeonggi-do 456-756, Republic of Korea; 3 College of Pharmacy, Dongguk University, Seoul 100-715, Republic of Korea

## Abstract

**Background:**

A major problem with the use of current chemotherapy regimens for several cancers, including breast cancer, is development of intrinsic or acquired drug resistance, which results in disease recurrence and metastasis. However, the mechanisms underlying this drug resistance are unknown. To study the molecular mechanisms underlying the invasive and metastatic activities of drug-resistant cancer cells, we generated a doxorubicin-resistant MCF-7 breast cancer cell line (MCF-7/DOX).

**Methods:**

We used MTT (3-(4,5-dimethylthiazol-2-yl)-2,5-diphenyltetrazolium bromide) assays, flow cytometry assays, DNA fragmentation assays, Western blot analysis, cell invasion assays, small interfering RNA (siRNA) transfection, reverse transcription-polymerase chain reaction, experimental lung metastasis models, and gelatin and fibrinogen/plasminogen zymography to study the molecular mechanism of metastatic activities in MCF-7/DOX cells.

**Results:**

We found that MCF-7/DOX acquired invasive activities. In addition, Western blot analysis showed increased expression of epidermal growth factor receptor (EGFR) and Cox-2 in MCF-7/DOX cells. Inhibition of Cox-2, phosphoinositide 3-kinase (PI3K)/Akt, or mitogen-activated protein kinase (MAPK) pathways effectively inhibited the invasive activities of MCF-7/DOX cells. Gelatin and fibrinogen/plasminogen zymography analysis showed that the enzymatic activities of matrix metalloproteinase-2 (MMP-2), MMP-9, and urokinase-type plasminogen activator were markedly higher in MCF-7/DOX cells than in the MCF-7 cells. *In vitro *invasion assays and mouse models of lung metastasis demonstrated that MCF-7/DOX cells acquired invasive abilities. Using siRNAs and agonists specific for prostaglandin E (EP) receptors, we found that EP1 and EP3 played important roles in the invasiveness of MCF-7/DOX cells.

**Conclusions:**

We found that the invasive activity of MCF-7/DOX cells is mediated by Cox-2, which is induced by the EGFR-activated PI3K/Akt and MAPK pathways. In addition, EP1 and EP3 are important in the Cox-2-induced invasion of MCF-7/DOX cells. Therefore, not only Cox-2 but also EP1 and EP3 could be important targets for chemosensitization and inhibition of metastasis in breast cancers that are resistant to chemotherapy.

## Background

Breast cancer is the most common malignancy and a major cause of death among women in the Western world [1]. Many anticancer agents, including 5-fluorouracil, cyclophosphamide, and monoclonal antibodies such as trastuzumab, have shown efficacy in extending the survival of breast cancer patients; however, the mechanisms by which these agents inhibit breast cancer progression are not clearly understood. Although many promising anticancer agents have been developed and show potential in preclinical trials, classic chemotherapeutic agents such as doxorubicin are still widely used in patients [2].

A major problem with the use of chemotherapy to treat many cancers (including breast cancer) is intrinsic or acquired drug resistance, which results in disease recurrence and metastasis. Recent results from several laboratories have investigated the mechanism by which breast cancer cells become resistant to doxorubicin, as well as the molecular profile of breast cancer cells that are resistant to doxorubicin [3,4]. Bcl-xl is responsible for acquisition of resistance to chemotherapeutic agents such as doxorubicin, leading to decreased apoptosis and increased survival of breast cancer cells [5,6]. Furthermore, recent evidence has suggested that the ability of tumor cells to acquire an aggressive phenotype may result from accumulation of genetic alterations conferred by extended survival [7,8].

Cox-2 is involved in the inflammatory response and its expression is commonly upregulated in human cancers; therefore, Cox-2 has been suggested to play a major role in tumorigenesis [9,10]. Recent studies have reported that Cox-2 plays a key role as a regulator of chemotherapy resistance in cancer. Cox-2 expression has been reported to be indicative of an aggressive breast cancer phenotype that is resistant to doxorubicin [11]. For example, drug-resistant cell lines that overexpress P-glycoprotein 170 (MDR1/Pgp170) also have significantly upregulated Cox-2 expression, indicating a strong correlation between Cox-2 expression and resistance to chemotherapy in breast cancer cell lines [12]. In addition, selective inhibition of Cox-2 suppresses the invasion activity of oral squamous cells through downregulation of a matrix metalloproteinase-2 (MMP-2)-activating mechanism [13]. Cox-2 overexpression in human breast cancer cells enhances their motility and invasiveness [14]. Furthermore, Cox-2 overexpression in human breast cancers correlates with several clinical parameters that are characteristic of aggressive breast disease [15,16]. Inhibitors that are selective for Cox-2 have been developed as anti-inflammatory agents and also show effective anticancer properties in breast cancer patients at risk for disease recurrence. Furthermore, inhibition of Cox-2 has a significant effect on the drug resistance and metastatic potential of cancer cells [17]. Knocking down Cox-2 using small interfering RNA (siRNA) or Cox-2 inhibitors suppresses cell growth and invasion and enhances the chemosensitivity of cancers, including breast cancer [18-20].

Several lines of evidence have suggested that metastasis may be enhanced by an ability to resist apoptosis and highly metastatic cancer cells exhibit greater survival ability and resistance to apoptosis than poorly metastatic cells [21,22]. Therefore, cancer cells may acquire invasive and metastatic properties during the process of becoming resistant, a mechanism that remains poorly understood. To identify genes associated with the invasive and metastatic activities of drug-resistant cells, we analyzed changes in gene expression in doxorubicin-resistant MCF-7 breast cancer cells (MCF-7/DOX) that we established using DNA array analysis. We observed invasive activities related to high expression of Cox-2 in MCF-7/DOX cells.

Having identified Cox-2 as an important regulator of the invasiveness of MCF-7/DOX cells, we next asked which upstream pathway modulates the expression of Cox-2 and how the invasive activities increased doxorubicin-resistant cancer in this study.

## Methods

### Animals, cells, and materials

Female 6-week-old Balb/c nude mice were purchased from Charles River Laboratories (Wilmington, MA, USA). The human breast cancer cell lines MDA-MB-231, MCF-7, and T-47D were obtained from the American Type Culture Collection (Manassas, VA, USA). MCF-7/DOX cells were derived from MCF-7 cells by continuous culture in the presence of doxorubicin (Sigma-Aldrich, St. Louis, MO, USA) for more than 3 months. Exposure of MCF-7 cells to stepwise increasing concentrations (0.1-1 μM) of doxorubicin resulted in the selection of doxorubicin-resistant MCF-7/DOX cells. Exposure to doxorubicin was terminated 4 days prior to the experiments. Cells were cultured in Dulbecco's modified Eagle's medium (DMEM) supplemented with 10% fetal bovine serum (FBS) and penicillin/streptomycin (Invitrogen, Grand Island, NY, USA). Cell culture inserts incorporating polyethylene terephthalate membranes (6.4-mm diameter, 8- μm pore size) and 24-well plates for invasion assays were purchased from Costar (Cambridge, MA, USA).

We obtained MTT [3-(4,5-dimethylthazol-2-yl)-2,5-diphenyltetrazolium bromide] from Sigma-Aldrich. The phosphoinositide 3-kinase (PI3K) inhibitor LY294002 and mitogen-activated protein kinase (MAPK) inhibitor U0126 were purchased from Calbiochem-Novabiochem (La Jolla, CA, USA). Sulprostone, 17-phenyl trinor Prostaglandin E_2 _(17-PT-PGE_2_), Prostaglandin E_2 _(PGE_2_), and the Cox-2 inhibitor NS398 were purchased from Cayman Chemical (Ann Arbor, MI, USA). Epidermal growth factor (EGF) was purchased from R&D Systems Inc. (Minneapolis, MN, USA). Gefitinib was purchased from Biaffin GmbH & Co KG (Kassel, Germany). Gelatin, fibrinogen, and plasminogen were obtained from Sigma-Aldrich. Antibodies against ERK1, Cox-2, and actin were purchased from Santa Cruz Biotechnology (Santa Cruz, CA, USA). Antibodies against the EGF receptor (EGFR), pAkt, Akt, phosphorylated extracellular signal regulated kinase 1/2 (pERK1/2), pEGFR, poly(ADP-ribose) polymerase (PARP), and tubulin were purchased from Cell Signaling Technology (Danvers, MA, USA). Doxorubicin was purchased from Sigma-Aldrich and dissolved in phosphate-buffered saline (PBS) at various concentrations to establish dose responses. Synthetic siRNAs targeting EGFR, prostaglandin E receptor 1 (EP1), and EP3 were purchased from Bioneer (Seoul, Korea) and have the following sequences: EGFR (5'-GGCACGAGUAACAAGCUCA-3'); EP1 (5'-GUCGGUAUCAUGGUGGUGU-3'); and EP3 (5'-GUCAUCGUCGUGUACCUGU-3').

### MTT assay

The inhibitory effect of doxorubicin, the Cox-2 inhibitor NS398, and the PI3K inhibitor LY294002 on growth of MCF-7 and MCF-7/DOX cell lines was determined using the MTT assay. Cells were plated onto 96-well plates (3 × 10^3 ^cells/well) and cultured in medium with or without various concentrations of doxorubicin, NS398, LY294002, gefitinib, and U0126. The cells then were grown for an additional total incubation of 24 or 72 h.

### Flow cytometry assay

Cells were harvested, washed, fixed with paraformaldehyde and 70% ethanol, and stained using an APO-BRDU kit (Biovision, Inc., Mountain View, CA, USA) according to the manufacturer's protocol. Flow cytometric analysis was performed using a BD FACS Calibur flow cytometer (BD Biosciences, San Jose, CA, USA) equipped with a 488-nm argon-ion laser. Approximately 10,000 events (cells) were evaluated for each sample.

### Western blot analysis

Total cell extracts were prepared from human breast cancer cells treated with various drugs as indicated. Preparation of whole-cell lysates, protein quantification, gel electrophoresis, and Western blotting were performed as described elsewhere [23]. Protein concentrations were measured using the bicinchoninic acid protein assay (Pierce Biotechnology, Rockford, IL, USA), as described in the manufacturer's protocol. Equivalent amounts of protein from cell lysates or conditioned media (CM) from each treatment group were resolved by sodium dodecyl sulfate-polyacrylamide gel electrophoresis (SDS-PAGE) and immunoblotted with primary antibodies. Bands were detected using ECL Western blotting detection reagents from GE Healthcare (Chalfont St. Giles, United Kingdom).

### Invasion assays

*In vitro *invasion assays were performed as described elsewhere [24]. Briefly, CM obtained by culturing Wi38 fibroblasts for 18 h in DMEM with 10% FBS was placed into the lower chamber of each well as a chemoattractant. The upper chamber contained 1 × 10^5 ^MCF-7/DOX cells incubated in media alone or in the presence of NS398, LY294002, gefitinib, or U0126 for 18 h. Cells were fixed and stained with hematoxylin and eosin. Filters were cut out and mounted on glass slides for cell counting. Cells from the entire membrane field were counted. All experiments were repeated at least three times.

### siRNA transfection

MCF-7/DOX cells were transfected with siRNA using Lipofectamine RNAiMAX (Invitrogen, Carlsbad, CA, USA). The siRNA-transfected cells were incubated for 48 h and harvested for Western blot analysis.

### Reverse transcription-polymerase chain reaction (RT-PCR)

Total RNA was isolated from MCF-7/DOX cells using Trizol reagent (Invitrogen). cDNAs synthesized from 1 μg of total RNA were used as templates in a 50- μl reaction using the TaqMan RT reagents according to the manufacturer's protocol (Applied Biosystems, Foster City, CA). RT-PCR was performed to amplify genes using a cDNA template corresponding to gene-specific primer sets. The primer sequences used are as follows: EP1 (forward 5'-TCGGCCTCCACCTTCTTTGGC-3' and reverse 5'-CTGGCGCAGTAGGATGTACAC-3'), EP2 (forward 5'-GTCATGTTCTCGGCCGGGGTG-3' and reverse 5'-GAGGACTGAACGCATTAGTCT-3'), EP3 (forward 5'- CGCCGGGAGAGCAAGCGCAAG-3' and reverse 5'-GATGCGGCCCCACTGGGCACTGGA-3'), EP4 (forward 5'-ATCTTACTCATTGCCACC-3' and reverse 5'-TCTATTGCTTTACTGAGCAC-3'), urokinase-type plasminogen activator (uPA; forward 5'-CCAATTAGGAAGTGTAAGCAGC-3' and reverse 5'-GCCAAGAAAGGGACATCTATG-3'), MMP-2 (forward 5'-TCGCCCATCATCAAGTTC-3' and reverse 5'- GTGATCTGGTTCTTGTCC-3'), MMP-9 (forward 5'-AACCAATCTCACCGACAG-3' and reverse 5'-CAAAGGCGTCGTCAATCA-3'), β-actin (forward 5'-GTGGGGCGCCCCAGGCACCA-3' and reverse 5'-CTCCTTAATGTCACGCACGATTTC-3').

To avoid amplifying genomic DNA, gene primers were chosen from different exons. PCR was performed in a total reaction volume of 25 μl that contained 2 μl of cDNA solution and 0.2 μM of sense and antisense primers. The RT-PCR exponential phase was determined on cycles 28-33 to allow quantitative comparisons among the cDNAs amplified from identical reactions. The amplification products (8 μL) were resolved on a 2% agarose gel, stained with ethidium bromide, and visualized on a transilluminator and photographed.

### Experimental lung metastasis models

MCF-7 and MCF-7/DOX cells (3 × 10^6 ^cells in 100 μL PBS) were injected into the tail vein of Balb/c nude mice (MCF-7, n = 8; MCF-7/DOX, n = 9). Three months after injection, the animals were killed by CO_2 _inhalation and their lungs were excised. Lung tumor formation was observed and tumor nodules were counted under a dissecting microscope. All animal experiment procedures were approved by the Institutional Animal Care and Use Committee in Korea National Cancer Center.

### Gelatin and fibrinogen/plasminogen zymography

The proteolytic activity of MMP-2, MMP-9, and uPA in CM was analyzed by substrate-gel electrophoresis [25] using SDS-PAGE gels containing 0.2% (m/v) gelatin or 0.12% (m/v) fibrinogen and plasminogen (0.01 NIH unit/mL). CM from each treatment group was concentrated using an Amicon Ultra-4 centrifugal device (Millipore, Bedford, MA, USA) and loaded onto gels. After electrophoresis, the gels were washed with 2.5% Triton X-100 and incubated overnight in zymogram incubation buffer (50 mM Tris-HCl, 0.15 M NaCl, 10 mM CaCl_2_, and 0.02% NaN_3_) at 37°C. Clear bands indicative of gelatinolytic activity were visualized by staining the gels with Coomassie blue.

### Gene expression analyses from whole genome

Total RNA was isolated and purified from MCF-7 and MCF-7/DOX cells using the TRIzol reagent (Invitrogen) and RNease Mini kit (QIAGEN). Of those, 500 ng RNA was biotinylated and amplified using the Illumina TotalPrep RNA Amplification Kit (Ambion, TX) according to the manufacturer's instructions. The cRNA yield was measured using RiboGreen RNA quantitation kit (Invitrogen), and 750 ng of the cRNA sample was hybridized on a human HT-12 expression bead chip (Illumina, CA) for profiling 48,804 transcripts per sample. Bead chips were stained with streptavidin and scanned using an Illumina BeadArray Reader. BeadStudio V3 was used to quantile-normalize the data.

To find doxorubicin-resistant phenotype associated genes, we applied expression data to search and include genes with significant difference in expression levels between MCF-7 and MCF-7/DOX. Gene sets with 2-fold or more difference in mRNA level and *p *value cutoff (0.05) are presented in Table 1.

**Table 1:** Differentially requlated genes in MCF-7/DOX cells

Gene symbol	Name	Fold change
*EGFR*	Epidermal growth factor receptor	19.079
*TWIST1*	Twist transcription factor	8.661
*MMP9*	Matrix metallopeptidase 9	5.890
*PLAU*	Plasminogen activator, urokinase	159.280
*TIMP3*	TIMP metallopeptidase inhibitor3	0.020
*CDH2*	N-cadherin	94.160
*CDH1*	E-cadherin	0.001

### Statistical analysis

The effect of doxorubicin or NS398 on breast cancer cell proliferation was analyzed using one-way ANOVA followed by Turkey's multiple test (SPSS version 10; SPSS, Chicago, IL, USA). The data of *in vitro *cancer cell invasion and tumor incidence in the mice were analyzed using Student's *t*-test (Microsoft Excel software version 2007; Microsoft Corporation).

## Results

### Resistance of MCF-7/DOX cells to doxorubicin

**Figure 1:**
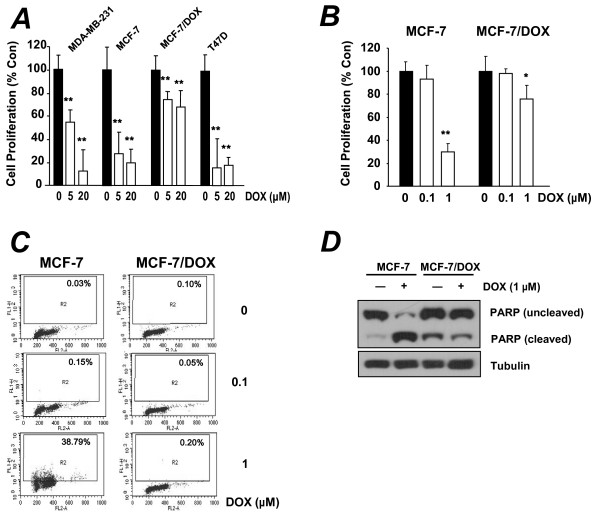
**Resistance to doxorubicin in MCF-7/DOX cells**. (**A, B**) Cell proliferation in MDA-MB-231, MCF-7, MCF-7/DOX, and T-47D breast cancer cells 72 h after treatment with doxorubicin at (**A**) high or (**B**) low concentration. (**C**) The number of apoptotic cells was counted by terminal deoxynucleotidyl transferase dUTP nick end labeling-based fluorescence-activated cell sorting analysis 48 h after doxorubicin treatment. (**D**) Western blot analysis of MCF-7 and MCF-7/DOX cells following treatment with doxorubicin for 48 h. Values shown are means ± standard deviation (SD). *P < 0.01; **P < 0.001 compared with the untreated group.

Doxorubicin is one of the most commonly used drugs in the treatment of cancer, but its inhibitory effect on cell proliferation varies in several cancer cell lines. Therefore, we investigated whether doxorubicin has the same antiproliferative effect in MDA-MB-231, MCF-7, MCF-7/DOX, and T-47D cell lines. The cells were treated with the indicated concentrations of doxorubicin for 72 h and cell proliferation was measured using the MTT assay. Doxorubicin inhibited cell proliferation in a concentration-dependent manner in MCF-7 and T47D cells, and to a lesser extent in MDA-MB-231 cells. By contrast, doxorubicin-mediated inhibition was significantly reduced in MCF-7/DOX cells (Figure 1A). We next measured the growth of MCF-7 and MCF-7/DOX cells at lower doxorubicin concentrations and MCF-7/DOX cells were consistently resistant to doxorubicin (Figure 1B).

We then tested whether the doxorubicin-mediated growth inhibition was mediated by apoptosis. After MCF-7 and MCF-7/DOX cells were treated with 1 μM doxorubicin for 48 h, terminal deoxynucleotidyl transferase-mediated dUTP nick end labeling-based fluorescence-activated cell sorting analysis showed that doxorubicin did not induce apoptosis in MCF-7/DOX cells; however, doxorubicin did induce apoptosis in MCF-7 cells (Figure 1C). We further confirmed the resistance of MCF-7/DOX cells to doxorubicin by Western blot analysis. Induction of PARP cleavage (a marker of apoptosis) in MCF-7 cells confirmed that doxorubicin induced apoptosis in these cells. However, PARP was not cleaved in MCF-7/DOX cells treated with doxorubicin (Figure 1D).

### Acquisition of invasive and metastatic properties in MCF-7/DOX cells

Intrinsic or acquired drug resistance results in disease recurrence and metastasis [26]. We analyzed changes in gene expression in doxorubicin-resistant MCF-7/DOX cells using DNA array analysis. Differentially expressed genes related with invasion are listed in Table 1.

**Figure 2:**
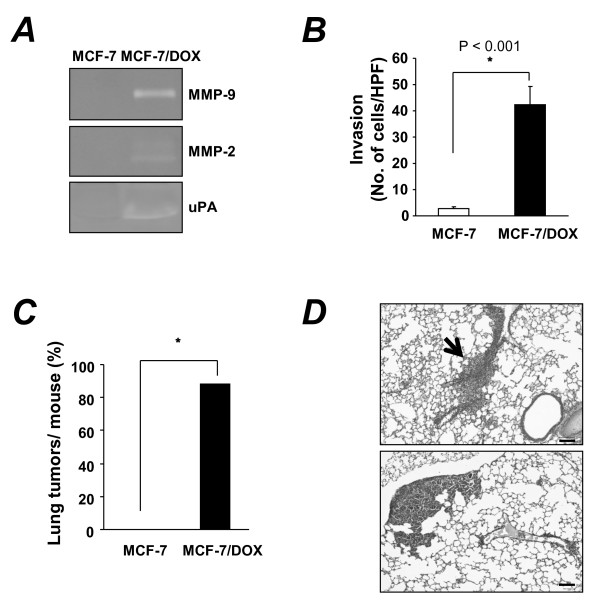
**Acquisition of invasive and metastatic properties by MCF-7/DOX cells**. (**A**) The activity of MMP-2, MMP-9, and uPA in MCF-7 and MCF-7/DOX cells was determined by gelatin or fibrinogen/plasminogen zymography. (**B**) An invasion assay was performed with MCF-7 and MCF-7/DOX cells. Invasion of MCF-7 and MCF-7/DOX cells through a transwell membrane coated with Matrigel was compared. *P < 0.001 (*t*-test). (**C**) Postmortem examination of the lungs of mice that were killed 3 months after injection of MCF-7 or MCF-7/DOX cells. Lung tissues were fixed in Bouin's solution, and the gross tumor nodules were counted. *P < 0.001 (Chi-square test). (**D**) Representative photograph of lung tumors from nude mice injected with MCF-7/DOX cells. The arrow head indicates invading MCF-7/DOX cells in the lung of nude mice. Original magnification, × 100; Scale bar = 100 μm.

We next examined whether MCF-7/DOX cells acquired metastatic properties. First, we measured the enzymatic activities of MMP-9, MMP-2, and uPA in MCF-7 and MCF-7/DOX cells by gelatin and fibrinogen/plasminogen zymography. The enzymatic activities of MMP-2, MMP-9, and uPA were markedly higher in MCF-7/DOX cells than in non-invasive MCF-7 cells (Figure 2A). Increased levels of MMP-9 and MMP-2 expression have been correlated with an invasive phenotype of cancer cells [27]. Thus, we assessed the invasiveness of MCF-7 and MCF-7/DOX cells using *in vitro *invasion assays. As expected, the MCF-7/DOX cells were significantly more invasive than MCF-7 cells (Figure 2B). To test the invasive activity of MCF-7/DOX cells *in vivo*, we developed a breast cancer model of lung metastasis. Three months after injecting MCF-7/DOX cells through the tail veins of balb/c nude mice, the mice were killed, and the total number of visible lung tumor nodules per mouse was quantified under a stereomicroscope. Lung tumor nodules were present in the mice injected with MCF-7/DOX cells, while no mouse injected with MCF-7 cells had lung metastases (Figure 2C and 2D). These results suggest that MCF-7 cells obtained metastatic abilities after acquiring resistance to doxorubicin.

### Cox-2 mediates invasiveness of MCF-7/DOX cells

**Figure 3:**
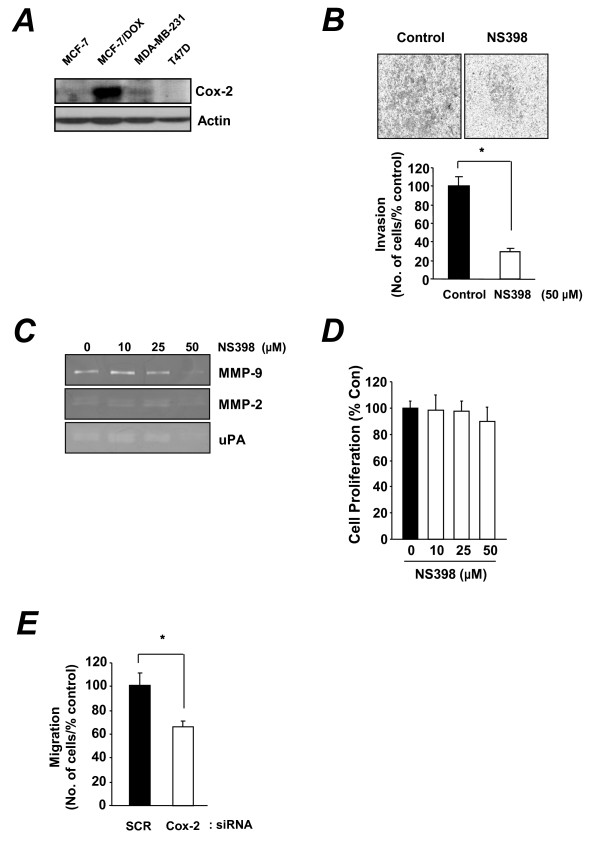
**The role of Cox-2 in invasiveness of MCF-7/DOX cells**. (**A**) Western blot analysis of Cox-2 expression in MDA-MB-231, MCF-7, MCF-7/DOX, and T-47D cells. (**B**) Invasion assay with MCF-7/DOX cells treated with 50 μM NS398 for 18 h. (**C**) The effect of NS398 on uPA, MMP-2, and MMP-9 activity in MCF-7/DOX cells was determined by gelatin or fibrinogen/plasminogen zymography. (**D**) Cell proliferation in MCF-7 and MCF-7/DOX cells treated with the indicated concentrations of NS398 for 72 h. *P < 0.01. (**E**) A cell migration assay with MCF-7/DOX cells transfected with Cox-2-specific siRNA.

Recent studies have reported that Cox-2 plays a key role as a regulator of chemotherapy resistance in cancer [28]. In addition, Cox-2 expression plays an important role in the metastatic and invasive abilities of cancer cells [29]. Selective inhibition of Cox-2 was shown to suppress the invasion of oral squamous cells by downregulating an MMP-2-activating mechanism [13]. Therefore, we tested whether invasiveness of MCF-7/DOX cells is related to Cox-2 expression. Western blot analysis showed a high basal level of Cox-2 in doxorubicin-resistant MCF-7/DOX cells and metastatic MDA-MB-231 cells (Figure 3A).

Recent studies have reported that Cox-2-overexpressing cells (MCF-7/Cox-2) demonstrate increased invasiveness [30]. Moreover, several studies have suggested that targeting Cox-2 may protect against development of invasive breast cancer [17-19]. Thus, we tested the effects of a Cox-2 inhibitor on invasion of MCF-7/DOX cells. Treatment of MCF-7/COX cells with the Cox-2 inhibitor NS398 decreased their invasive potential (Figure 3B), indicating that Cox-2 expression contributes to the invasive activity of MCF-7/DOX cells. We next determined the effects of the Cox-2 inhibitor NS398 on activities of MMP-9, MMP-2, and uPA secreted from MCF-7/DOX cells using plasminogen/fibrinogen and gelatin zymography assays. We found that the activity of MMP-9 and uPA was inhibited by 50 μM NS398, a concentration that did not affect MCF-7/DOX cell proliferation (Figure 3C and 3D). The effect of Cox-2 expression on invasiveness of MCF-7/DOX cells was confirmed by blocking Cox-2 expression using siRNA. Consistent with the results of the Cox-2 inhibitor experiment, transfection of siRNA targeting Cox-2 suppressed migration of MCF-7/DOX cells in an *in vitro *migration assay (Figure 3E).

### Effect of the EGFR pathway on Cox-2-mediated invasion of MCF-7/DOX cells

**Figure 4:**
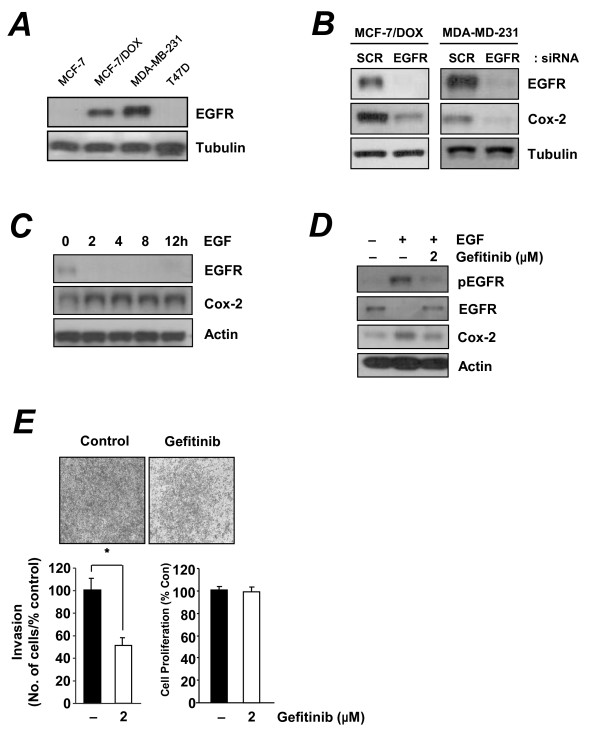
**Effect of the EGFR pathway on Cox-2 mediated invasiveness of MCF-7/DOX cells**. (**A**) Western blot analysis of EGFR expression in MDA-MB-231, MCF-7, MCF-7/DOX, and T-47D cells. (**B**) Western blot analysis of MCF-7/DOX and MDA-MB-231 cells that were transfected with control or EGFR siRNA. (**C**) Western blots of lysates from MCF-7/DOX cells treated with EGF (100 ng/mL) for the indicated times (0-12 h). (**D**) Western blot analysis from MCF-7/DOX cells that were treated with gefitinib (2 μM) for 24 h And EGF (100 ng/mL) was added for an additional 8 h. (**E**) Cell invasion assay of MCF-7/DOX cells treated with 2 μM gefitinib treatment for 18 h. Cell proliferation was not affected by treatment with 2 μM gefitinib for 24 h. *P < 0.01 (*t*-test).

Having identified Cox-2 as an important regulator of invasiveness of MCF-7/DOX cells, we next asked which upstream pathway modulates the expression of Cox-2 in this cell line. Because EGFR has been reported to regulate Cox-2 expression [31], we hypothesized that activation of the EGFR pathway may induce Cox-2 expression. First, we examined the basal expression level of EGFR in a subset of breast cancer cell lines. Western blot analysis showed high levels of EGFR expression in the doxorubicin-resistant MCF-7/DOX and MDA-MB-231 cells, which were invasive and had elevated Cox-2 expression (Figure 4A).

We next tested the role of the EGFR pathway on induction of Cox-2 expression by treating cells with EGF and blocking these pathways using EGFR-specific siRNA. Cox-2 expression was markedly suppressed when both MCF-7/DOX and MDA-MB-231 cells were transfected with EGFR-specific siRNAs (Figure 4B). In addition, Western blot analyses of MCF-7/DOX cells revealed that Cox-2 expression was induced within 2 h of EGF treatment (Figure 4C). We further confirmed that blocking the EGFR pathway suppressed Cox-2 expression using a selective EGFR tyrosine kinase inhibitor, gefitinib. Treating cells for 24 h with gefitinib effectively suppressed EGF-induced Cox-2 expression in MCF-7/DOX cells (Figure 4D). We then tested the effects of the EGFR inhibitor on invasion of MCF-7/DOX cells. Gefitinib decreased the invasive potential of MCF-7/DOX cells (Figure 4E), indicating that EGFR contributes to invasiveness of MCF-7/DOX cells by upregulating Cox-2.

### Effect of EGFR on PI3K/Akt- and MAPK-mediated Cox-2 expression in MCF-7/DOX cells

**Figure 5:**
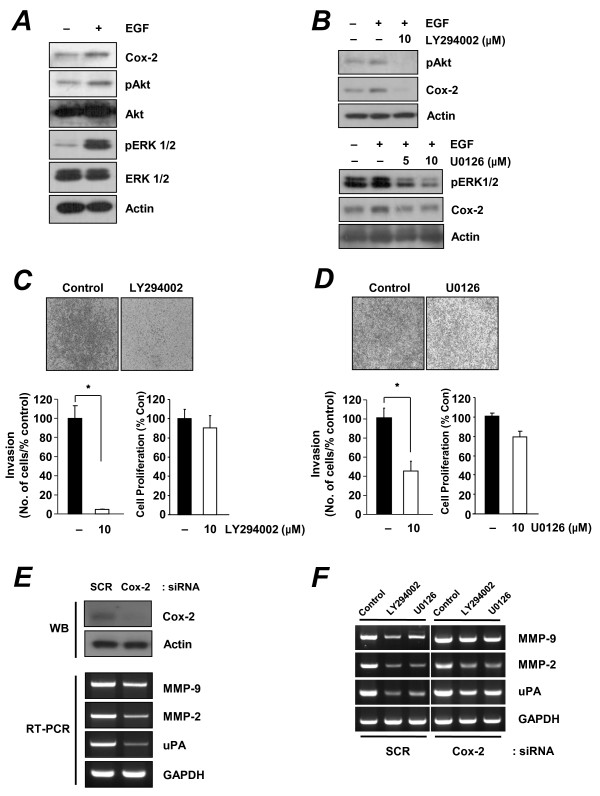
**Effect of PI3K/Akt and MAPK pathways on Cox-2 expression in MCF-7/DOX cells**. (**A**) Western blot analysis in MCF-7/DOX cells treated with EGF (100 ng/mL) for 8 h. (**B**) Western blot analysis of MCF-7/DOX cells that were treated with LY294002 (10 μM) or U0126 for 9 h. EGF (100 ng/mL) was added 8 h before cell harvest. (**C, D**) Invasion assay of MCF-7/DOX cells treated with either (**C**) 10 μM LY294002 or (**D**) U0126 for 18 h. Cell proliferation was also measured with using the MTT assay 24 h after treatment. *P < 0.01 (*t*-test). (**E**) Western blot and RT-PCR analysis of MCF-7/DOX cells that were transfected with control or Cox-2 siRNA. (**F**) Semiquantitative RT-PCR analysis of uPA, MMP-2, and MMP-9 in MCF-7/DOX cells transfected with siRNAs specific for Cox-2 and treated with LY294002 (10 μM) or U0126 (10 μM) for 48 h.

Because EGFR controls several other pathways, such as the PI3K/Akt and MAPK pathways [32,33], we next investigated which downstream pathway was involved in EGFR-mediated Cox-2 expression. As expected, treating MCF-7/DOX cells with EGF for 8 h to induce Cox-2 expression activated both the PI3K/Akt and MAPK pathways (Figure 5A). To confirm the role of the PI3K/Akt and MAPK pathways in Cox-2 expression, we studied the effect of the PI3K/Akt inhibitor LY294002 and the MAPK inhibitor U0126 on EGF-induced expression of pAkt and Cox-2 in MCF-7/DOX cells. Western blot analysis showed that LY294002 and U0126 dramatically suppressed activation of pAkt and pERK1/2, respectively, and downregulated EGF-induced Cox-2 expression (Figure 5B). To investigate the role of the PI3K/Akt pathway in invasiveness of MCF-7/DOX cells, we determined whether blocking the PI3K/Akt pathway would inhibit invasion of MCF-7/DOX cells in an *in vitro *invasion assay. Blocking the PI3K/Akt pathway with LY294002 or the MAPK pathway with U0126 dramatically inhibited invasion of MCF-7/DOX cells, but did not affect proliferation of the cells (Figure 5C and 5D). Because we wondered whether PI3K/Akt or MAPK signaling alone not through Cox-2/PGE_2 _can increase expression of MMP-2, MMP-9, and uPA, we tested MMP-2, MMP-9, and uPA mRNA expression in MCF-7/DOX cells transfected with siRNA specific for Cox-2 (Figure 5E). Transfection with siRNAs targeting Cox-2 specifically inhibited MMP-2, MMP-9, or uPA RNA expression in MCF-7/DOX cells, whereas control scrambled siRNA demonstrated no effect (Figure 5F). Expression of MMP-2, MMP-9 and uPA mRNA was suppressed by the PI3K/Akt inhibitor LY294002 and the MAPK inhibitor U0126 in MCF-7/DOX cells transfected with control siRNA; however, their expressions were less affected by either LY294002 or U0126 in MCF-7/DOX cells transfected with Cox-2 siRNA (Figure 5F).

### Role of EP1 and EP3 receptors in Cox-2 mediated invasion of MCF-7/DOX cells

**Figure 6:**
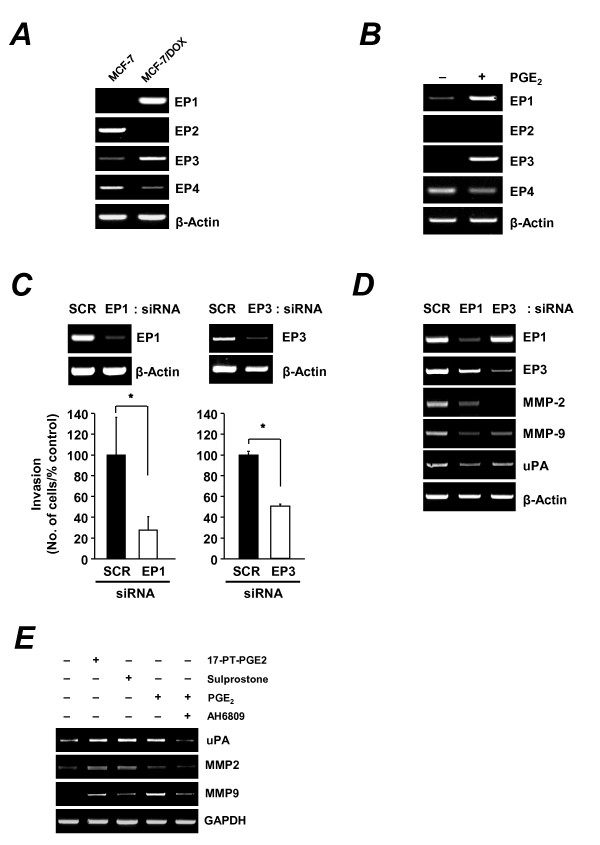
**The role of the EP1 and EP3 receptors in Cox-2-mediated invasion of MCF-7/DOX cells**. (**A**) The expression of EP receptor mRNAs was determined by RT-PCR in MCF-7 and MCF-7/DOX cells. (**B**) MCF-7/DOX cells were serum starved for 12 h, treated with PGE_2 _(10 μM) for 24 h, and the expression of EP receptor mRNAs was determined by RT-PCR. (**C**) MCF-7/DOX cells were transfected with siRNAs specific for EP1 or EP3 and analyzed using cell invasion assays. Cells were treated with PGE_2 _(10 μM) to activate invasion of MCF-7/DOX cells. (**D**) Semiquantitative RT-PCR analysis of uPA, MMP-2, and MMP-9 in MCF-7/DOX cells transfected with siRNAs specific for EP1 or EP3. (**E**) MCF-7/DOX cells were starved in serum-free medium for 18 h and pretreated with 17-PT-PGE_2_, sulprostone, or PGE_2 _for 13 h from 1 h before AH6809 treatment (10 μM, 12 h). MMP-2, MMP-9, and uPA expression were determined by semiquantitative RT-PCR analysis.

PGE_2_, which is produced by Cox-2, exerts its effect by binding to specific prostanoid receptors (EP1, EP2, EP3, and EP4), which are a family of G-protein-coupled receptors [34]. Thus, we investigated the role of EP receptors in the Cox-2-mediated invasion in MCF-7/DOX cells. First, we compared the basal expression levels of the EP receptors in MCF-7 and MCF-7/DOX cells by RT-PCR. We found that EP1 and EP3 mRNAs were upregulated, while EP2 and EP4 mRNAs were downregulated in MCF-7/DOX cells (Figure 6A). Similarly, specific induction of EP1 and EP3 mRNA, but not EP2 and EP4 mRNA was observed in MCF-7/DOX cells treated with PGE_2 _for 24 h (Figure 6B). To determine which EP receptor regulates invasive activities of MCF-7/DOX cells, we blocked expression of either EP1 or EP3 with gene-specific siRNAs and performed an invasion assay. Invasive activities induced by PGE_2 _in MCF-7/DOX cells were inhibited by suppression of either EP1 or EP3 expression (Figure 6C). We further confirmed the effect of EP1 and EP3 on uPA, MMP-2, and MMP-9 expression by measuring the expression levels of uPA, MMP-2, and MMP-9 after blocking EP1 and EP3 expression with gene-specific siRNAs. RT-PCR data showed that expression of MMP-2 and MMP-9 were reduced when expression of EP1 or EP3 was inhibited (Figure 6D). To determine which EP receptor regulates invasive activities of MCF-7/DOX cells, cells were treated with EP1- or EP3-specific agonists and MMP-2, MMP-9 and uPA mRNA expression was examined by RT-PCR. Only EP1/EP3 receptor or EP3 agonists significantly increased MMP-2, MMP-9, and uPA mRNA expression. Furthermore, treatment with the EP1 receptor antagonist AH6809 effectively attenuated MMP-2, MMP-9, and uPA mRNA expression by PGE_2 _in MCF-7/DOX cells (Figure 6E).

## Discussion

Chemotherapy plays an important role in the treatment of breast cancer; however, long-term treatment often results in chemoresistance, leading to disease recurrence and metastasis [18-20]. To study the molecular mechanisms underlying invasive and metastatic activities in drug-resistant cancer cells, we generated the doxorubicin-resistant MCF-7 breast cancer cell line MCF-7/DOX. We found that MCF-7/DOX breast cancer cells displayed enhanced metastatic and invasive behavior both in *in vitro *cell invasion assays and *in vivo *in a mouse lung tumor model. We demonstrated that invasiveness of MCF-7/DOX cells resulted from Cox-2 activation, which was induced by either the EGFR-activated PI3K/Akt or MAPK pathway. Inhibiting either Cox-2 or the PI3K/Akt pathway effectively inhibited the invasiveness of MCF-7/DOX cells.

Cox-2 was coexpressed with EGFR in human colorectal cancer and bronchial adenocarcinomas [35,36] and induced in a human glioma cell line [31]. We investigated the mechanisms by which EGFR signaling regulates Cox-2 expression. The EGFR pathway controls several pathways, including the PI3K/Akt and MAPK pathways [32]. Our data showed that, in MCF-7/DOX cells, Cox-2 expression was regulated by both the PI3K/Akt and Ras/Raf/MAPK pathways through EGFR signaling. Western blot analysis showed that, in MCF-7/DOX and MDA-MB-231 cells, Cox-2 expression was reduced when EGFR expression was blocked by an EGFR-specific siRNA. In addition, the EGFR inhibitor gefitinib significantly suppressed EGF-induced Cox-2 expression and invasion of MCF-7/DOX cells. These data provide evidence that Cox-2 expression induced by the EGFR pathway is associated with invasiveness of MCF-7/DOX cells.

PGE_2_, the major end product of Cox-2 activation, is also known to activate EGFR through various pathways [37]. Therefore to clarify whether PGE_2 _signaling through EPs promotes the PI3K/Akt or MAPK pathway-mediated invasion primarily, we evaluated the effect of EP1- or EP3-specific agonists or EP inhibitor on the PI3K/Akt or MAPK pathways, but we found that PGE_2 _signaling through EPs didn't affect PI3K/Akt and MAPK pathway in the MCF-7/DOX cells (data not shown).

Recent studies have shown that Cox-2 mRNA and protein expression in several cancer cell lines are regulated by the insulin-like growth factor (IGF)-1R/PI3K and nuclear factor-kappa B/nuclear factor of kappa light polypeptide gene enhancer in B-cells inhibitor pathways [38,39]. In addition to the PI3K/Akt pathway, the Ras/Raf/MAPK pathway is also a downstream transducer of IGF-1R signaling. The IGF-1R signaling pathway plays a major role in cell proliferation, apoptosis, invasion, and angiogenesis. Moreover, IGF-1R has been shown to upregulate Cox-2 mRNA expression and PGE_2 _synthesis in cancer cells [40]. Although we found that IGF-1R expression was neither increased nor constitutively activated in MCF-7/DOX cells, activation of the IGF-1R pathway may still contribute to Cox-2 expression and our efforts are ongoing to determine any further possibility.

Treating cells with EGF also increased pAkt and pERK1/2 expression in MCF-7/DOX cells. To investigate the role of the PI3K/Akt pathway in Cox-2 expression, we studied the effect of the PI3K/Akt inhibitor LY294002 on EGF-induced pAkt and Cox-2 expression. Western blot analysis showed that LY294002 dramatically suppressed pAkt activation and Cox-2 expression induced by EGF in MCF-7/DOX cells.

Because Cox-2 exerts its effects by producing PGE_2_, which binds to specific EP receptors [34], we investigated the role of specific EP receptors in Cox-2-mediated invasion of MCF-7/DOX cells. PGE_2 _treatment induced expression of the EP1 and EP3 receptors, suggesting that these two receptors are likely involved in the invasiveness by MCF-7/DOX cells. Both EP1 and EP3 receptors played an important role in Cox-2 induced invasion of MCF-7/DOX cells. We showed that selective inhibition of EP1 and EP3 using siRNAs inhibited PGE_2_-induced invasion of MCF-7/DOX cells, as well as expression of MMP-2 and MMP-9. A previous study showed increased Cox-2 expression in patients with poorly differentiated breast cancer and poor clinical outcomes for patients treated with doxorubicin [11]. However, the expression pattern of EP receptors has never been studied in breast cancer. Therefore, our findings are the first to suggest a pivotal role for the EP1 and EP3 receptors in doxorubicin-resistant breast cancer cells.

## Conclusions

Together, we have demonstrated that invasiveness of MCF-7/DOX cells results from Cox-2 activation, which is induced by either the EGFR-activated PI3K/Akt or MAPK pathway. Inhibiting Cox-2 or the EGFR pathway effectively inhibited invasiveness of MCF-7/DOX cells. We also found that the EP receptors EP1 and EP3 are important for Cox-2-induced invasion of MCF-7/DOX cells. Therefore, not only Cox-2 but also EP1 and EP3 could be important targets for chemosensitizing and inhibiting metastasis in chemotherapy-resistant breast cancers.

## Competing interests

The authors declare that they have no competing interests.

## Authors' contributions

All authors participated in design of the study. JHK performed the experimental work and wrote the manuscript. KHS, KCJ, SK, CC, and CHL contributed to data analysis and interpretation. SHO conceived of the study, participated in the experimental design, and helped to draft the manuscript. All authors read and approved the final manuscript.

## Pre-publication history

The pre-publication history for this paper can be accessed here:

http://www.biomedcentral.com/1471-2407/11/334/prepub
